# Enhanced OER Performance and Dynamic Transition of Surface Reconstruction in LaNiO_3_ Thin Films with Nanoparticles Decoration

**DOI:** 10.1002/advs.202207128

**Published:** 2023-02-24

**Authors:** Huan Liu, Rongrong Xie, Qixiang Wang, Jiale Han, Yue Han, Jie Wang, Hong Fang, Ji Qi, Meng Ding, Weixiao Ji, Bin He, Weiming Lü

**Affiliations:** ^1^ Spintronics Institute School of Physics and Technology University of Jinan Jinan 250022 P. R. China; ^2^ Functional Materials and Acousto‐Optic Instruments Institute School of Instrumentation Science and Engineering Harbin Institute of Technology Harbin 150080 P. R. China; ^3^ School of Physics and Technology University of Jinan Jinan 250022 P. R. China

**Keywords:** nanoparticles, oxygen evolution reaction, single‐crystal thin films, surface reconstruction, transition metal oxides

## Abstract

In an electrocatalytic process, the cognition of the active phase in a catalyst has been regarded as one of the most vital issues, which not only boosts the fundamental understanding of the reaction procedure but also guides the engineering and design for further promising catalysts. Here, based on the oxygen evolution reaction (OER), the stepwise evolution of the dominant active phase is demonstrated in the LaNiO_3_ (LNO) catalyst once the single‐crystal thin film is decorated by LNO nanoparticles. It is found that the OER performance can be dramatically improved by this decoration, and the catalytic current density at 1.65 V can be enhanced by ≈1000% via ≈10^9^ cm^−2^ nanoparticle adhesion after extracting the contribution of surface enlargement. Most importantly, a transition of the active phase from LNO to NiOOH via surface reconstruction with the density of LNO nanoparticles is demonstrated. Several mechanisms in terms of this active phase transition are discussed involving lattice orientation‐induced change of the surface energy profile, the lattice oxygen participation, and the A/B‐site ions leaching during OER cycles. This study suggests that the active phases in transition metal‐based OER catalysts can transform with morphology, which should be corresponding to distinct engineering strategies.

## Graphical abstract

The dramatic enhancement of OER performance in LaNiO_3_ (LNO) single‐crystal catalysts has been achieved by the controllable decoration of surface LNO nanoparticles. Meanwhile, stepwise evolution of the active phase from LNO to NiOOH with nanoparticle adhesion is demonstrated, which indicates a morphology dependence of the dynamic surface reconstruction.

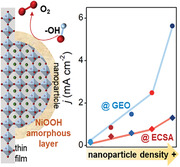

## 1 Introduction

The nanoparticlized catalyst has greatly boosted the performance of an electrochemistry process, such as oxygen evolution reaction (OER) owing to its specific chemical properties and extremely enlarged effective surface area.^[^ ^1^, ^2^, ^3^, ^4^, ^5^, ^6^ ^]^ In the transition metal oxide (TMO), one of the most promising OER catalysts, the reduction of dimensionality can offer a unique surface energy profile and abundant dangling bonds by size confinement, this makes the non‐bulk electronic structure dominates in the OER process.^[^ ^7^, ^8^, ^9^, ^10^ ^]^ Thus, some emergent OER phenomena are frequently accompanied by nanoparticlizing the TMO catalyst and cannot be understood only by an enlarged surface. As we all know, some perovskite TMO electrocatalysts could undergo surface reconstruction under the OER condition to generate new transition metal oxyhydroxide active species.^[^ ^11^, ^12^, ^13^, ^14^, ^15^ ^]^ And in principle, the characteristic of the dimensionality reduction in the nanoparticles could cause the differentiation of surface reconstruction making the active phase different in some TMO catalysts with morphological change. Sun et al. found that LaNiO_3_ (LNO) nanoparticles display a NiOOH re‐deposition surface layer once the LNO undergoes the OER process in an alkaline electrolyte, and the active phase reconstruction was promoted via A‐site Ce doping.^[^ ^16^ ^]^ Zhao et al. deposited FeOOH clusters on the surface of LNO powder to reduce the surface amorphization and stabilize the structure of LNO.^[^ ^17^ ^]^ However, Liu et al., Song et al., and Wang et al. reported that the LNO single‐crystal thin film can exhibit excellent stability during the OER cycles, indicating that few transitions of the surface active phase occur.^[^ ^18^, ^19^, ^20^, ^21^ ^]^ These observations present that there is an inconsistent cognization of the real active phase between the TMO and the oxhydryl radical oxide, although they are separately identified experimentally. However, it remained unclear as to how the nanoparticle properties of the TMO direct the surface structure transformation during the OER process. This uncertainty greatly confuses the effective design of promising OER catalysts as varied engineering strategies should be adopted on discrepant active phases. Thus, it is highly desirable to develop nanoparticlized strategies for introducing the dynamic structure evolution without additional chemical offset and creating the coherent correlation of dimensionality‐activity in the OER process. Yet it is known the isolation and the subsequent catalytic measurement of a single nanoparticle built a huge technological barrier. So the engineering of a catalyst with a continuous designable density and distribution of nanoparticles is highly desired to approach its fundamentality.

Perovskite LNO is a superior OER active catalyst with good electrical conductivity, which could exclude the effect of conducting additives on the OER performance. Besides, the energy profile of 3*d* electrons can be efficiently engineered by strain and stoichiometry design to promote OER activity. Here, in this study, we have fabricated the LNO single‐crystal thin films with surface‐decorated LNO nanoparticles which are controllable in density. The connection or correlation of these nanoparticles can be negligible as their average distance is larger than ≈150 nm. Then we comprehensively compare the performance of the OER on these different nanoparticle configurations. It is found that the current density at the potential of 1.65 V normalized by ECSA increases ≈10 times via the introduction of the nanoparticles on the surface of the LNO thin film, which indicates that the OER activity improvement could be attributed to not only the surface area enlargement from the nanoparticle but also the surface reconstruction. Furthermore, we have found the surface reconstruction of a NiOOH amorphous layer is deposited on the nanoparticles, and reconstruction potential decreased from ≈1.45 to ≈1.40 V with the increase of the nanoparticle densities. Our investigation provides new insight into the active phase in LNO with morphology benefiting further the design of promising OER catalysts.

## 2 Results and Discussion

**Figure 1:**
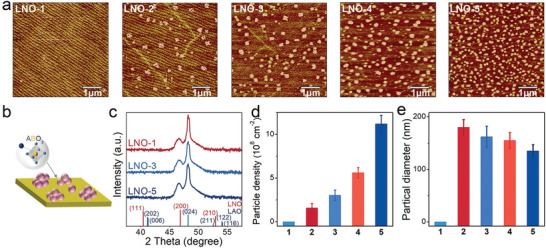
a) The AFM images of LNO thin films with different surface nanoparticle densities. b) Schematic illustration of the nanoparticles formed over the surface of LNO thin film. c) The XRD pattern of LNO thin films with different surface nanoparticle densities. d) The surface nanoparticle densities of the LNO thin films. e) The surface nanoparticle diameters of the LNO thin films.

A series of epitaxial single‐crystal LNO thin films with different surface nanoparticle densities were prepared by pulsed laser deposition (PLD) on (001)‐oriented LaAlO_3_ (LAO) substrates. The different surface nanoparticle densities were controlled by varying the growth oxygen pressures through the values of 100, 130, 160, 190, and 210 mTorr, which are denoted as LNO‐1, LNO‐2, LNO‐3, LNO‐4, and LNO‐5, respectively. Moreover, all samples were annealed under the same high oxygen pressure to eliminate oxygen deficiencies for the oxygen stoichiometry consistency on the surface. In **Figure** **1**a, the AFM images show the LNO thin films with different surface nanoparticle densities, which increase with the growth oxygen pressure. The oxygen atmosphere over optimal conditions can enhance the plasma scattering during the PLD deposition, this makes the 3D growth model pronounced to a nanoparticle tendency. Among them, the LNO‐1 possesses an atomically flat surface, and almost no surface nanoparticles are observed. However, from LNO‐2 to LNO‐5, the nanoparticles are well independently distributed on the surfaces of the other LNO thin films and exhibit a mostly spherical shape. Figure S1, Supporting Information presents the SEM images of different surface nanoparticle densities, which are consistent with the AFM result. The schematic illustration of the nanoparticles formed over the surface of the LNO thin film is displayed in Figure 1b,c shows the XRD pattern of the LNO thin films with different surface nanoparticle densities. All samples remain strictly epitaxial along with the (001) orientation and without any noticeable peaks of impurity structural phases. The mean nanoparticle densities and diameters of the LNO thin films are summarized in Figure 1d,e, which are determined from the AFM images (Figure 1a,d displays the mean surface nanoparticle densities of 0 cm^−2^ for LNO‐1, (1.6 ± 0.5)×10^8^ cm^−2^ for LNO‐2, (3.0 ± 0.5)×10^8^ cm^−2^ for LNO‐3, (5.5 ± 0.5)×10^8^ cm^−2^ for LNO‐4, and (11.2 ± 1)×10^8^ cm^−2^ for LNO‐5. It is seen that the densities of the surface nanoparticles can be well controlled by the growth oxygen pressure. In Figure 1e, from LNO‐1 to LNO‐5, the nanoparticle diameters are gradually smaller with the increasing nanoparticle densities, which are 0, 180 ± 15, 162 ± 20, 155 ± 15, and 135 ± 12 nm, respectively. This implies that the density of the point defect on the surface may increase with the oxygen pressure, and the oversaturated growth oxygen atmosphere could induce cationic defects. Anyhow, we have achieved the LNO film with homogeneous and engineerable nanoparticle adhesion, this surface decoration enables us to comprehensively reveal the possible active phase evolution of the LNO catalyst in OER.

**Figure 2:**
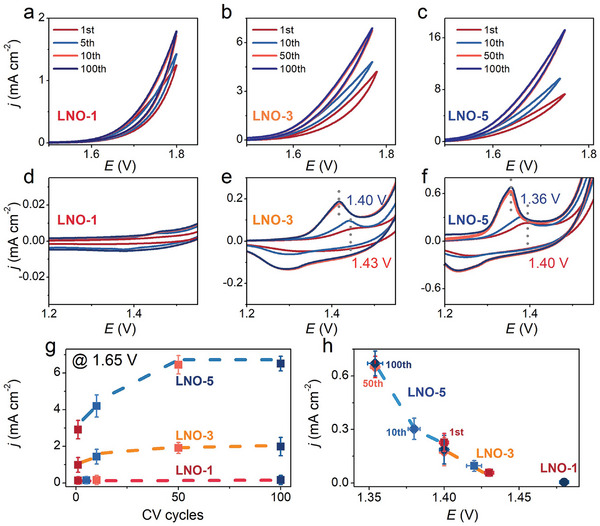
a) The 1st, 5th, 10th, and 100th CV scans of LNO‐1. The 1st, 10^th^, 50^th^, and 100^th^ CV scans of b) LNO‐3 and c) LNO‐5. d) The 1^st^, 5^th^,10^th^, and 100^th^ CV scans at the capacitive region of LNO‐1. The 1^st^, 10^th^, 50^th^, and 100^th^ CV scans at the capacitive region of e) LNO‐3 and f) LNO‐5. g) Changes of the current densities at the potential of 1.65 V during the 100 CV cycles. h) Changes in the oxidation peak positions before and after 10, 50, and 100 CV cycles.

The OER catalytic activities of the LNO thin films are evaluated by cyclic voltammetry (CV) in 1 mol L^−1^ KOH electrolyte. **Figure** **2**a–c exhibit the CV cycles of LNO‐1, LNO‐3, and LNO‐5 at a scan rate of 5 mV s^−1^, and the amplified capacitive regions are shown in Figure 2d–f, respectively. Both the OER and capacitive current increase with the number of CV cycles. Figure 2g directly presents the increase of the current density at the potential of 1.65 V during 100 CV cycles. The current densities of LNO‐3 and LNO‐5, decorated by nanoparticles, continuously increase during CV cycling and approach stability after ≈50 cycles, yielding ≈100% (from 0.99 to 1.98 mA cm^−2^) and 125% (from 2.91 to 6.51 mA cm^−2^) greater OER current than that in the first cycle. In contrast, the OER current of LNO‐1 increases by only ≈25% (from 0.14 to 0.17 mA cm^−2^) and reached stability during the first 10 cycles. Previous reports present that some perovskite oxides may exhibit an appearance of the oxyhydroxide layer on the surface after OER measurements, here this layer is amorphous and may be more OER active.^[^ ^22^, ^23^, ^24^ ^]^ Further, in Figure 2e,f, the existence of significant oxidation peaks of LNO‐3 and LNO‐5 indicates chemical reconstruction reaction occurs before the OER process. Along with the redox peaks being more prominent, the OER activities of the LNO samples gradually increase during successive cycling, which indicates that the major improvement of the OER activity could be explained by the reconstruction of a new surface NiOOH amorphous layer. In addition, oxidation peak positions shift towards lower potentials with the increase of nanoparticle densities. The extracted oxidation peak variations are summarized in Figure 2h. To be specific, the oxidation potentials appear between ≈1.43–1.40 V for LNO‐3, and LNO‐5 exhibits a lower oxidation potential between ≈1.40–1.36 V from the 1^st^ cycle to the 100^th^ cycle, which suggests the structural reconstruction evolution depends on the surface nanoparticle density. This suggests a facilitated effect of the surface nanoparticles by the surface structural reconstruction from the perovskite phase to the active NiOOH amorphous layer.

**Figure 3:**
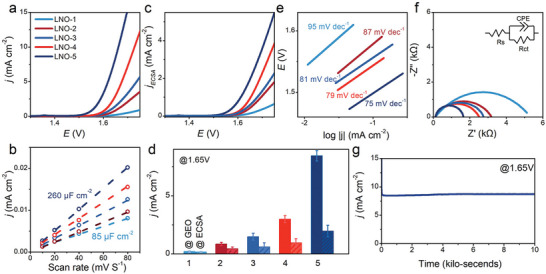
a) LSV curves after 100 CV cycles, b) *C*_dl_, c) LSV curves normalized by ECSA, d) the summarized current density normalized by geometry area and ECSA at the potential of 1.65 V, e) Tafel plots, f) EIS Nyquist plots of the LNO thin films with different surface nanoparticle densities. g) Chronoamperometry curve at the potential of 1.65 V of the LNO‐5.

The linear sweep voltammetry (LSV) curves of the LNO with different nanoparticle densities after cycling 100 times in 1 mol L^−1^ KOH are shown in **Figure** **3**a. The OER activities are enhanced with the increase of the surface nanoparticle density. LNO‐1 with no surface nanoparticles exhibits the most limited catalytic activity (0.19 mA cm^−2^ at 1.65 V), and the current density of LNO‐5 reaches 5.7 mA cm^−2^ at the potential of 1.65 V, which is improved by around 30 times compared with LNO‐1. Furthermore, the electrochemical double‐layer capacitance (*C*_dl_) is estimated by the CV method in the non‐faraday current region to determine the electrochemical active surface area (ECSA). As shown in Figure 3b, the *C*_dl_ values are 85, 118, 150, 191, and 260 µF cm^−2^ for LNO‐1, LNO‐2, LNO‐3, LNO‐4, and LNO‐5, corresponding detailed CV curves can be found in Figure S2, Supporting Information. It is not far to seek that the improvement of the OER activity partially originates from the enlargement of ECSA owing to the increase of surface nanoparticle density. However, the OER activity from LNO‐1 to LNO‐5 is enhanced almost 30 times with only three times increase in ECSA (from 85 to 260 µF cm^−2^). To better understand the reason for the enhanced OER activities of the LNO thin films decorated by different surface nanoparticle densities, the LSV curves normalized by ECSA are shown in Figure 3c. It is found that the OER activities normalized by ECSA are also enhanced with the increase of the surface nanoparticle densities, similar to Figure 3a. The current density normalized by geometry area (GEO) and ECSA at the potential of 1.65 V as a function of the surface nanoparticle density are summarized in Figure 3d. The current density normalized by ECSA increases ≈10 times from 0.13 to 1.31 mA cm^−2^ via the introduction of the nanoparticles on the surface of the LNO film, which proves that the OER activity improvement could be attributed to not only the enlargement of the ECSA but also the surface reconstruction of an active NiOOH amorphous layer. Moreover, Figure S3, Supporting Information shows the curves of experimentally measured and theoretically calculated O_2_ amount of the LNO‐5 under a steady‐state potential of 1.65 V for 100 min. Accordingly, the LNO‐5 exhibits a high Faradaic efficiency of 96.8%. However, we note that the Faradaic efficiency for the first 20 min is only 89.8%, smaller than that after 20 min, which could attribute to the gradual formation of oxyhydroxide species on the catalyst surface at the first 20 min. The Tafel plots, potential versus the logarithm of current density, are shown in Figure 3e. The Tafel slopes are 95, 87, 81, 79, and 75 mV dec^−1^ for the samples of LNO‐1, LNO‐2, LNO‐3, LNO‐4, and LNO‐5, respectively. Obviously, LNO‐5 with the maximum surface nanoparticle density obtains the smallest Tafel slope, indicating the most rapid OER reaction kinetics. In Figure 3f, electrochemical impedance spectroscopy (EIS) at the potential of 1.65 V is measured and fitted using an equivalent circuit (in the inset). The charge transfer resistance (*R*_ct_) is extracted to be 5019.2, 3065.2, 2600.5, 2396.3, and 1532.1 Ω for the LNO‐1, LNO‐2, LNO‐3, LNO‐4, and LNO‐5, respectively. The result demonstrates that R_ct_ declines with the increase of surface nanoparticle density, which confirms that the surface nanoparticles provide enhanced charge transfer kinetics. The chronoamperometry curve of LNO‐5 at 1.65 V is shown in Figure 3g, suggesting that the sample undergoing surface reconstruction still has excellent durability.

**Figure 4:**
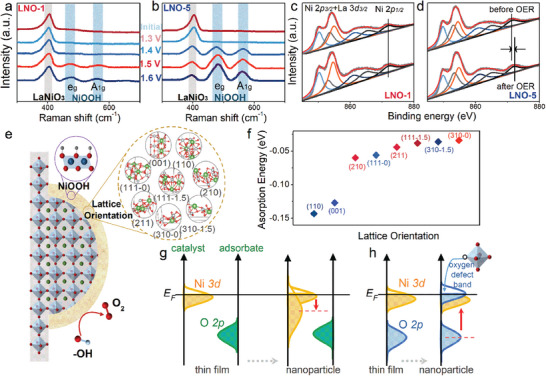
In situ Raman spectra of a) LNO‐1 and b) LNO‐5 at various potentials during the OER process. Ni 2p XPS spectra of c) LNO‐1 and d) LNO‐5. e) Schematic illustration of the surface reconstruction for LNO thin film decorated by nanoparticles. f) The adsorption energy of OOH for OER in different lattice orientations. g,h) Schematic illustrations of the Ni 3*d* band and O 2*p*‐band for LNO thin film and nanoparticle.

In **Figure** **4**a,b, the in situ electrochemical Raman spectroscopy is employed during the OER process to deeply investigate the changes in the surface structure of LNO with different nanoparticle densities. The peaks at around ≈402 cm^−1^ belong to E_g_ modes for the LNO phase.^[^ ^25^ ^]^ For LNO‐5 decorated by nanoparticles, the LNO peak gradually decreases, while the new peaks at ≈470 and ≈560 cm^−1^, typical vibration peaks of Ni‐O (E_g_ and A_1g_ modes) in NiOOH structure,^[^ ^26^, ^27^ ^]^ emerge at ≈1.4 V and progressively grow with the increase of the applied potential during OER, indicating that the LNO‐5 undergoes the surface reconstruction at the potential above ≈1.4 V. However, the Raman peaks of LNO‐1 at ≈470 and ≈560 cm^−1^ just weakly appear when the applied potentials are above 1.5 V and the peaks at 402 cm^−1^ are almost invariable, implying that the surface structural evolution is insignificant and the LNO perovskite phase on the surface is stable. These results are consistent with the CV measurements in Figure 2. This further confirmed that the strong surface reconstruction prefers to occur in the region of nanoparticles on the surface, rather than the atomically flat surface which maintains a stable LNO structure during OER.

XPS measurement was performed for the LNO with different surface nanoparticle densities before and after OER cycles. Figure 4c,d shows the Ni 2*p* XPS spectra of LNO‐1 and LNO‐5. Upon deconvolution, it is seen that the Ni 2*p*_3/2_ peaks (from 545 to 557 eV) are strongly overlapped with the existing La 3*d*_3/2_ satellite peak. The peaks at 861 and 865 eV are assigned to the Ni 2*p*_3/2_ satellite peaks. So the Ni 2*p*_1/2_ peaks are chosen to analyze the valence state of Ni in LNO.^[^ ^28^, ^29^, ^30^ ^]^ Ni 2*p*_1/2_ peaks of LNO‐1 remain consistent, while the peak position of LNO‐5 slightly shifts toward higher binding energy (from ≈871.5 to ≈872.5 eV) after the OER cycles, suggesting an increase in Ni valence.^[^ ^31^ ^]^ According to the Ni valence of NiOOH is between +3 and +4,^[^ ^32^, ^33^ ^]^ it is indicated that the formation of the NiOOH amorphous layer of LNO‐5 under the OER condition, but hardly in LNO‐1. O 1s XPS spectra of LNO‐1 and LNO‐5 before and after OER cycles are shown in Figure S5, Supporting Information. Upon deconvolution, the first peak at low binding energy is assigned to the lattice oxide ions from the perovskite oxide and the additional peaks at high binding energy are assigned to the O_2_^2−^/O^−^, hydroxyl groups, and chemisorbed oxygen.^[^ ^34^, ^35^, ^36^ ^]^ After OER cycles, the O 1s spectrum of LNO‐1 is not obviously changed. However, for LNO‐5, the peaks from the perovskite lattice oxygen greatly reduce, and the peak intensity of hydroxides increases. These results again confirm that the observed activity change with different nanoparticle densities is more than a result of surface area increase, and nanoparticles are more conducive to promoting the active phase reconstruction than atomically flat surfaces. Moreover, the XRD patterns of LNO‐5 are essentially identical before and after the OER indicating the overall crystalline phase of LNO remains unchanged after the OER in Figure S4, Supporting Information.

Our surface LNO nanoparticle decoration provides a stepwise evolution as a function of the morphology. Structurally the nanoparticles exhibit abundant lattice orientations and dangling bonds, in addition, a serious offset of the stoichiometry can introduce emergent strain conditions.^[^ ^37^, ^38^, ^39^ ^]^ These factors could greatly drive the surface energy profile of a nanoparticle catalyst away from the single‐crystal one which owns the ideal ordering of surface atom arrangement. So, it is inevitable to have a special surface chemical environment to present the unique unbalanced ability of adsorption/adhesion of the reaction intermediates while plentiful lattice orientations with defects in a nanoparticle are exposed. Then the deposited amorphous NiOOH, as the new active phase, could develop on the nanoparticle rapidly rather than on the surface of a single‐crystal thin film. In Figure 4f, we have plotted the OOH absorption ability of the varied lattice orientation of LNO via density functional theory (DFT) calculations, it can be seen that the surface energy characteristics show a strong dependence on the lattice orientation. Moreover, the nanoparticle LNO surface can serve an energy shift of Ni 3*d* as well as the O 2*p* bands exhibiting a non‐bulk energy profile, as shown in Figure 4g. The chemisorption can be changed once the lift of the Ni 3*d* band occurs, which optimizes the alignment with the O 2*p* band of electrolyte. In this situation, the NiOOH may be preferred to be adhesive on the surface of the nanoparticle rather than the thin film.

Furthermore, as shown in Figure S6a, Supporting Information, an enormous number of oxygen defects also exist on the surface of the nanoparticle, and the oxygen vacancies increase with the nanoparticle densities. Then the relationship between the oxygen vacancy content and the reconstruction potential from XPS and CV is shown in Figure S6b, Supporting Information. There is a linear dependence and it is found that the potential can be decreased by ≈0.05 V after introducing ≈5% oxygen vacancies. This could be attributed to an extra oxygen bond at the Fermi level (*E_F_*) of LNO caused by these charged defects, as shown in Figure 4h. Then the O 2*p* band center close to the *E_F_* could activate the lattice oxygen in the OER process by improving the participation of lattice oxygen, which promotes the formation of the surface NiOOH amorphous layer driven by the lattice oxygen evolution reaction.^[^ ^40^, ^41^, ^42^ ^]^ According to some literature, the formation of the NiOOH amorphous layer is usually accompanied by La and Ni leaching from LNO.^[^ ^8^, ^12^, ^16^, ^17^ ^]^ Then the leached Ni ions are re‐electrodeposition on the surface of perovskite as NiOOH in the OER cycles. It is imagable that the leaching of A/B‐site cations is determined by the stability of the ion in a compound, and the rich defects on the surface of nanoparticle LNO can facilitate this exsolution. Thus a clear transition of the active phases in LNO thin film and nanoparticle can be observed.

Anyhow, an elaborate evolution of the active phases is demonstrated in our LNO thin film and the ones with nanoparticle decoration, although the development of NiOOH could not be quantitatively determined. Thus varied prioritization schemes for high‐performance and stable OER catalysts should be adopted while the dynamic transition of surface reconstruction emerges with the morphological change. The *e*_g_ electron engineering seems more effective in single‐crystal thin films via the tunability of the adsorption/desorption ability of water intermediates.^[^ ^43^, ^44^, ^45^ ^]^ The morphological dressing in nanoparticle or nanoporous OER catalysts could be more promising to amplify the advantages of the unique surface characteristics and avoid the possible passivation during the active phase transition in OER cycles.

## 3 Conclusion

In summary, by decorating LNO nanoparticles on the surface of the LNO single‐crystal thin films, we have demonstrated the stepwise evolution of the dominant active phase with the morphology of the catalyst. By the introduction of the nanoparticles on the surface of the LNO thin film, the current density at 1.65 V normalized by ECSA increases ≈10 times than the pristine single‐crystal LNO thin film, which is attributed to the transition of the main active phase from LNO to NiOOH amorphous layer caused by surface reconstruction. Our investigation not only demonstrates the transition of the active phase with morphology during the OER process but also presents a new promising strategy for adjusting surface reconstruction by engineering morphology.

## 4 Experimental Section

### Sample Preparation

The PLD technique was used to grow the epitaxial LNO films on single‐crystal LAO (001) substrates. The substrate temperature was set to 700 °C. To adjust the nanoparticle densities, the growth oxygen pressures were changed between 100 and 210 mTorr, as specified in the main text. After each growth, the sample was annealed at the deposition temperature for 30 min under an oxygen pressure of 21 Torr to eliminate oxygen deficiencies for the same surface stoichiometry. Then the as‐prepared samples were cooled to room temperature at 5 °C min^−1^.

### Characterizations

X‐ray diffraction (XRD, Rigaku Smartlab) was used to examine the crystal structure. Atomic force microscopy (AFM, Asylum Research, MFP‐3D Origin+) was used to study the surface morphology. The X‐ray photoelectron spectroscopy (XPS, Thermo Fisher Scientific EscaLab 250) was performed using Al K*α* (hv = 1486.6 eV) radiation as the excitation source. Raman spectroscopy was carried out with a HORIBA HR800 Raman microscope with laser excitation at 532 nm.

### OER Measurements

The electrochemical characterization was performed in 1 mol L^−1^ KOH solution at room temperature. The counter and reference electrode is carbon rod and Hg/HgO electrode. LSV and CV curves were measured at scan rates of 1 and 5 mV s^−1^, respectively. The applied potentials were iR‐corrected with the measured current (*i*) and the ohmic electrolyte resistance (*R*_s_) determined via high‐frequency EIS measurements. All potentials were converted to a reversible hydrogen electrode (RHE): *E* versus RHE = *E* versus Hg/HgO + 0.098 + 0.059 × pH. All potentials mentioned in this study were versus RHE. More experimental details can be found in the reference.^[^ ^46^ ^]^

### DFT Calculations

The calculation was performed by the first‐principles method based on spin‐polarized density functional theory using the Vienna ab initio simulation package (VASP), in which the projector augmented wave (PAW) pseudopotential and the Perdew–Burke–Ernzerhof (PBE) exchange‐correlation functional were chosen. To describe the on‐site Coulomb strong correlation interaction of Ni‐3d electrons, the GGA+U method was employed with *U* = 5.1 eV. The plane wave truncation energy was set to 400 eV, the energy convergence criterion was set to 1e‐4 eV, and the optimization reached convergence when the force acting on each atom was less than 0.05 eV/Å. To avoid the interaction between adjacent supercells along direction c, a vacuum layer larger than 20 Å was added.

### Statistical Analysis

The data in Figure 1d,e was based on over three samples and presented in the form of the mean ± SD. The results presented in Figures 2g,h, and 3d were based on 15 samples and three sets of repeated experiments. Statistical analysis was performed using OriginPro 8.0 software.

## Conflict of Interest

The authors declare no conflict of interest.

## Supporting information

Supporting Information

## Data Availability

The data that support the findings of this study are available from the corresponding author upon reasonable request.
