# Supplementary material for: Enhanced OER Performance and Dynamic Transition of Surface Reconstruction in LaNiO3 Thin Films with Nanoparticles Decoration
Source: Adv Sci (Weinh). 2023 Feb 24;10(13):2207128. doi: 10.1002/advs.202207128 (PMC10161029; doi:10.1002/advs.202207128)
Supplement: Supplementary file 1 — Supporting Information [file ADVS-10-2207128-s001.pdf]

ADVANCED SCIENCE
Open Access

Supporting Information

for *Adv. Sci.*, DOI 10.1002/advs.202207128

Enhanced OER Performance and Dynamic Transition of Surface Reconstruction in $LaNiO_3$ Thin Films with Nanoparticles Decoration

Huan Liu, Rongrong Xie, Qixiang Wang, Jiale Han, Yue Han, Jie Wang, Hong Fang, Ji Qi, Meng Ding, Weixiao Ji, Bin He and Weiming Lü**

Supporting Information

Enhanced OER performance and dynamic transition of surface reconstruction in $LaNiO_3$ thin films with nanoparticles decoration

Huan Liu, Rongrong Xie, Qixiang Wang, Jiale Han, Yue Han, Jie Wang, Hong Fang, Ji Qi, Meng Ding, Weixiao Ji, Bin He, and Weiming Lü**

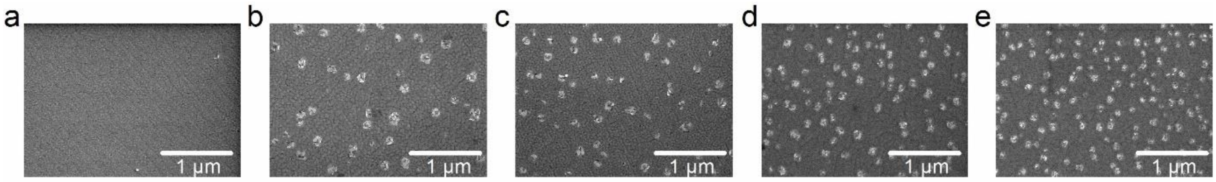

Figure S1. The SEM images of LNO thin films with different surface nanoparticle densities.

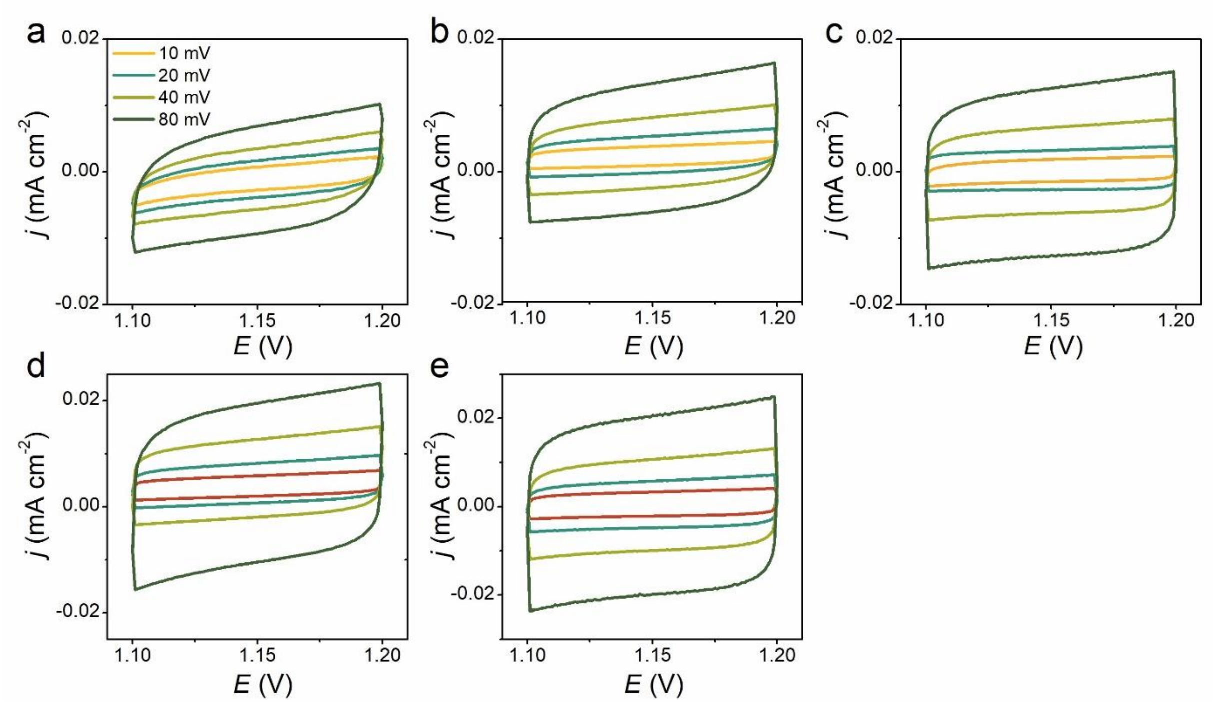

Figure S2. CV curves measured from 10 mV S^{-1} to 80 mV S^{-1} in the non-Faradaic potential region (1.1 to 1.2 V) for (a) LNO-1, (b) LNO-2, (c) LNO-3, (d) LNO-4 and (e) LNO-5. By

plotting the $\Delta j/2$ at 1.15 V against the scan rate, the linear slope is C_{dl}, which is proportional to the ECSA.

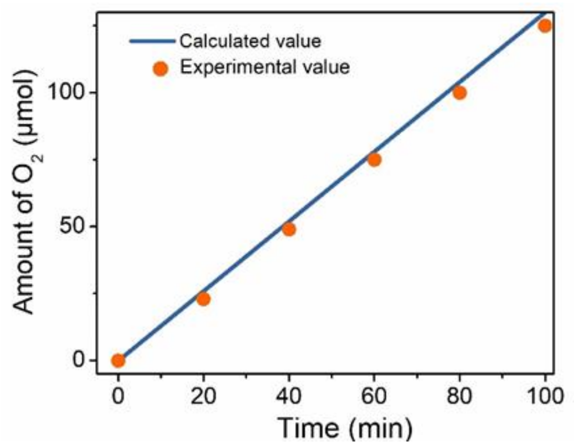

Figure S3. Faradaic efficiency of LNO-5 at the constant potential of 1.65 V.

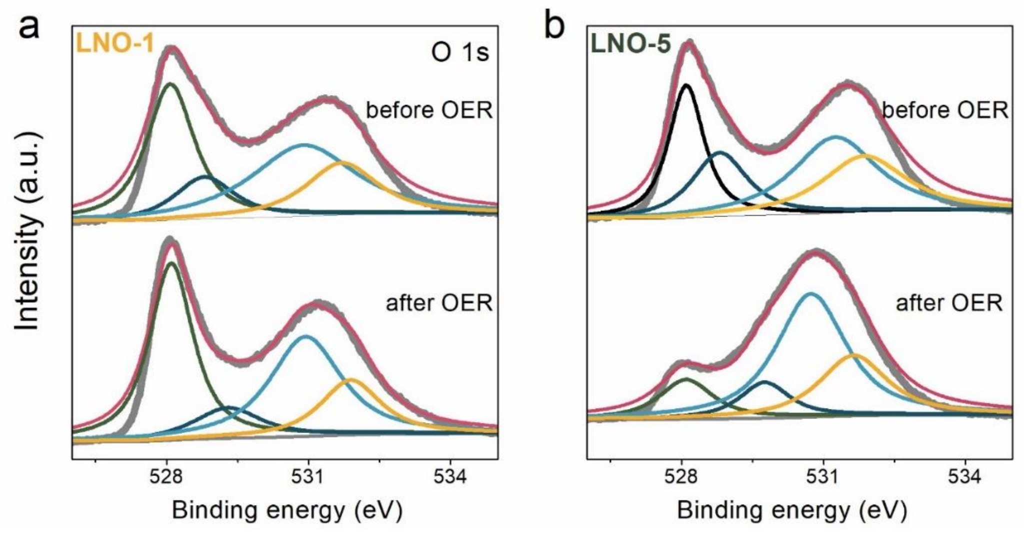

Figure S4. O 1s XPS spectra of (e) LNO-1 and (f) LNO-5.

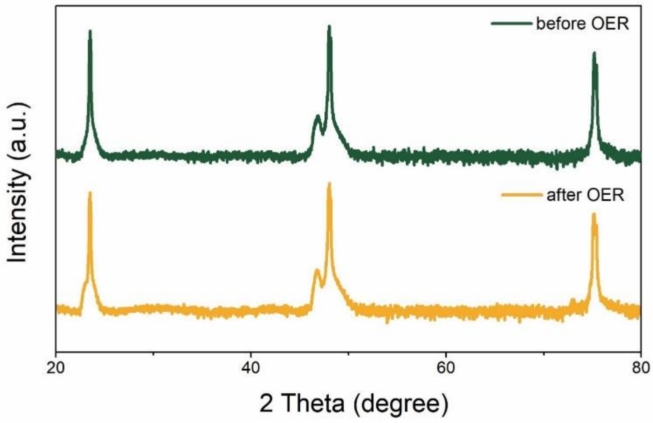

Figure S5. XRD patterns of LNO-5 before and after the 100 times OER cycles.

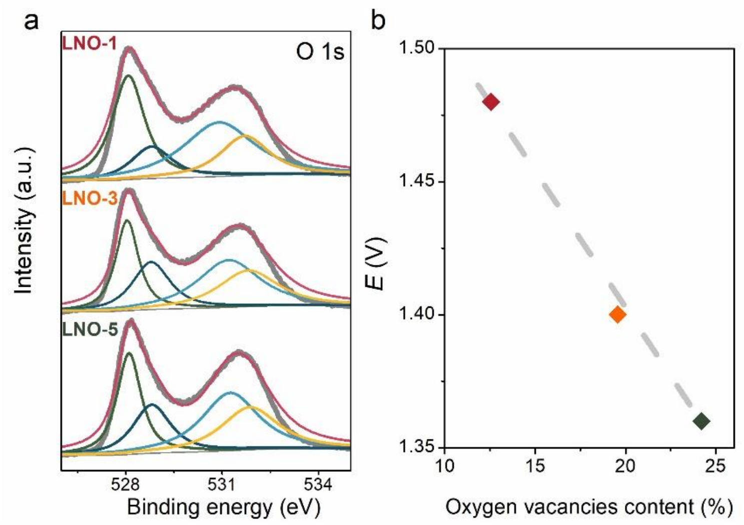

Figure S6. (a) O 1s XPS spectra of LNO-1, LNO-3, and LNO-5. (b) The reconstruction potentials versus the oxygen vacancies contents of LNO thin films with different surface nanoparticle densities.
